# Sensory integration deficits support a dimensional view of psychosis and are not limited to schizophrenia

**DOI:** 10.1038/tp.2017.69

**Published:** 2017-05-09

**Authors:** O Carter, D Bennett, T Nash, S Arnold, L Brown, R Y Cai, Z Allan, A Dluzniak, K McAnally, D Burr, S Sundram

**Affiliations:** 1 Melbourne School of Psychological Sciences, Faculty of Medicine, Dentistry and Health Sciences, University of Melbourne, Parkville, VIC, Australia; 2 Department of Psychiatry, University of Melbourne, Parkville, VIC, Australia; 3 Department of Psychology, University of Florence, Florence, Italy; 4 The Florey Institute of Neuroscience and Mental Health, Parkville, VIC, Australia; 5 Northern Psychiatry Research Centre, North Western Mental Health, Parkville, VIC, Australia; 6 Department of Psychiatry, School of Clinical Sciences, Monash University and Monash Health, Clayton, VIC, Australia

## Abstract

Visual dysfunction is commonplace in schizophrenia and occurs alongside cognitive, psychotic and affective symptoms of the disorder. Psychophysical evidence suggests that this dysfunction results from impairments in the integration of low-level neural signals into complex cortical representations, which may also be associated with symptom formation. Despite the symptoms of schizophrenia occurring in a range of disorders, the integration deficit has not been tested in broader patient populations. Moreover, it remains unclear whether such deficits generalize across other sensory modalities. The present study assessed patients with a range of psychotic and nonpsychotic disorders and healthy controls on visual contrast detection, visual motion integration, auditory tone detection and auditory tone integration. The sample comprised a total of 249 participants (schizophrenia spectrum disorder *n*=98; bipolar affective disorder *n*=35; major depression *n*=31; other psychiatric conditions *n*=31; and healthy controls *n*=54), of whom 178 completed one or more visual task and 71 completed auditory tasks. Compared with healthy controls and nonpsychotic patients, psychotic patients trans-diagnostically were impaired on both visual and auditory integration, but unimpaired in simple visual or auditory detection. Impairment in visual motion integration was correlated with the severity of positive symptoms, and could not be accounted for by a reduction in processing speed, inattention or medication effects. Our results demonstrate that impaired sensory integration is not specific to schizophrenia, as has previously been assumed. Instead, sensory deficits are closely related to the presence of positive symptoms independent of diagnosis. The finding that equivalent integrative sensory processing is impaired in audition is consistent with hypotheses that propose a generalized deficit of neural integration in psychotic disorders.

## Introduction

The discourse on the discreteness of psychotic disorders remains as contentious as when first proposed over 100 years ago.^1^ Research has traditionally focused primarily on individual disorders. This tendency has been particularly evident in schizophrenia research where the participant sample was frequently limited to strictly defined cases, whereas those with evidence of overlapping symptomatology were excluded. More recently, there have been moves away from categorical, towards more dimensional approaches to schizophrenia research in an attempt to define core pathology underlying observed dysfunction across a range of disorders.

Vision science is one area of schizophrenia research that has traditionally exemplified this tendency to limit main comparisons to those between individuals with strictly defined schizophrenia against either healthy controls or a second discrete clinical sample. Various sophisticated assessments of visual functioning in schizophrenia have clearly demonstrated that a wide range of deficits exist within this patient population using a range of both behavioral^2, 3, 4^ and neuroimaging measures.^5, 6^ Indeed, the systematic nature of these deficits has led many to suggest that they may elucidate the underlying pathophysiology of schizophrenia and provide a cheap, noninvasive biomarker for the disorder.^7, 8^ The selectivity of such deficits or their relationship to symptomatology, however, remains unclear with evidence suggesting a more complex picture. For example, studies using a static visual target detection task adapted from Banks and Prinzmetal,^9^ have demonstrated impaired perceptual organization in patients with schizophrenia that are actively symptomatic with poor premorbid function.^10^ In contrast, no such deficits were seen in ultra-high risk or first-episode psychosis.^11^

Motion perception is one deficit of visual function highlighted by a number of studies.^12, 13, 14, 15, 16, 17^ The specificity of these findings is itself informative, with individuals with schizophrenia showing impairments in tasks that involve the integration of multiple motion signals forming a ‘global’ pattern of motion across space.^13, 15, 18^ In contrast, the same participants show relatively normal sensitivity to individual ‘local’ motion signals.^13, 18^

A selective deficit in the motion integration task is consistent with a generalized neural integration deficit, which has been proposed to underlie schizophrenia and distinguish it from other psychiatric conditions—either through abnormalities in brain-wide functional connectivity^19, 20, 21^ or aberrant neurotransmitter system function.^22^ However, the symptoms most intuitively linked to a generalized functional integration deficit, such as hallucinations, delusions and conceptual disorganization, are also seen with other psychiatric disorders and suggest commonality across the psychosis spectrum.^23, 24^ To test this, we used an unbiased inclusive recruitment strategy aimed at assessing all eligible adults admitted to a psychiatric inpatient facility over approximately 24 months of testing.

It similarly follows that if a generalized functional integration deficit does exist, there should be no theoretical reason why the effects should be limited to the visual system. To determine whether a similar pattern of intact sensory detection and impaired integration extends beyond vision, we also assessed performance on a pair of tasks requiring simple auditory tone detection and subsequent integration of those signals to judge the relative coherence of frequency modulating sweeps. In addition, we designed the study to address several potential methodological confounds found in previous studies such as stimulus exposure duration and impaired sustained attention that could be relevant to patient populations.

## Materials and methods

### Participants

One-hundred and ninety-five adults (18–55 years) with a range of psychiatric disorders were recruited from an acute psychiatry inpatient unit in Melbourne, VIC, Australia. Fifty-four age-matched healthy controls were recruited from the hospital and university staff and students. The recruitment for different psychophysical tasks was sequential, such that different participant samples were recruited for different analyses, and no single participant completed all psychophysical tasks (clinical-demographic details are provided for the overall participant population in Table 1, diagnostic analysis and symptom correlation analysis in Supplementary Table 1, psychosis analyses in Supplementary Table 2 and control analyses in Supplementary Table 4). The healthy and patient sample was predominantly Caucasian with small numbers (<5%) of either East Asian or African.

Psychiatric diagnoses were made by a consultant psychiatrist and clinical team independent of investigators using the DSM-IV (Diagnostic and Statistical Manual of Mental Disorders 4th edition) criteria. Each diagnosis was then verified according to the DSM-IV criteria based on case file review by investigator (SS) blind to test data and confirmed using the Structured Clinical Interview for DSM-IV. Exclusion criteria were clinically documented or self-reported intellectual disability or intelligence quotient (IQ) <70 as measured by the National Adult Reading Test, history of traumatic brain injury, stroke or neurodegenerative disease. For healthy control participants, additional exclusion criteria were self-reported current use of psychotropic medication, illicit substance use within 2 weeks before testing, or personal history of psychiatric illness or substance use disorder. All the participants were conversant in English, had normal or corrected-to-normal vision/hearing, provided written informed consent and were financially compensated for their time. The study was approved by the Melbourne Health and University of Melbourne Human Research Ethics Committees.

### Participant information

Handedness was assessed using the Edinburgh Handedness Inventory^25^ and sociodemographic, family history and clinical—including medication (where relevant) data were collected. Premorbid IQ was estimated using the National Adult Reading Test.^26^ The majority of patients were also rated on the positive subscale of the PANSS (Positive and Negative Syndrome Scale)^27^ by trained raters (data were not collected for the negative subscale to reduce time demands for patients). All patients were receiving psychotropic medication during testing. For analysis purposes, antipsychotic dosages were converted to chlorpromazine equivalents, and benzodiazepine dosages were converted to diazepam equivalents.^28, 29^ The patients were classified as being psychotic if they had a current diagnosis of a schizophrenia spectrum disorder (SSD; schizophrenia, schizophreniform disorder or schizoaffective disorder), brief psychotic episode or drug-induced psychosis or had any other diagnosis (including bipolar disorder) and had psychotic features as indicated by a rating of ⩾3 on the delusions and/or hallucinations, and/or >4 on grandiosity or suspiciousness/persecution PANSS items. These criteria resulted in 152 patients being classified as psychotic and 32 as nonpsychotic from the 184 patients included in the visual and auditory psychosis analysis (see Supplementary Table 2 for demographic details and diagnostic breakdown within the psychosis groupings).

### Visual detection task

**Figure 1:**
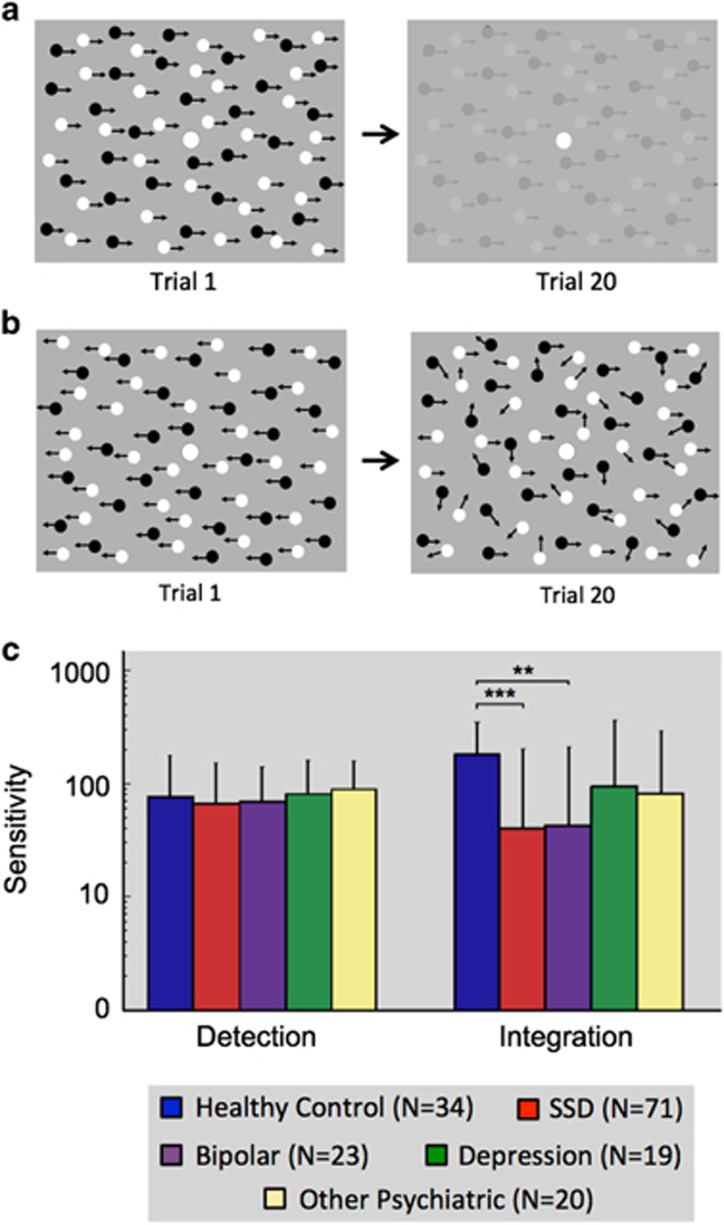
(**a**) Stimuli used for the visual detection and (**b**) integration task. In both instances, the participants reported whether the dots appeared to move to the left or right. Task difficulty was increased by either reducing the black/white contrast relative to the gray background (detection) or the ratio of coherent versus randomly moving dots (integration). (**c**) Mean sensitivity for different diagnostic groups across visual tasks. The results show no difference between groups in the detection task but significant impairment in integration for both the schizophrenia spectrum disorder (SSD; ***P<0.001) and bipolar (**P<0.01) patients relative to healthy controls. Error bars represent 95% confidence intervals.

Local motion detection was assessed by measuring contrast sensitivity to detect the direction of motion of a cloud of dots. Stimulus was a 9 × 9 cm array (9° × 9° visual angle) comprising 300 small dots (diameter 0.2 cm/0.2° visual angle) half black and half white, on a gray background. On each trial, subjects fixated a central white dot (0.5 cm/0.5° visual angle), observing the whole dot-pattern move horizontally left or right with 100% coherence and a velocity of 10° s^−1^ (see Figure 1a). Contrast began at 100% and then varied to home in near threshold following two independent adaptive QUEST algorithms. Sensitivity (inverse of threshold) was calculated offline with cumulative Gaussian functions to estimate 75% correct performance. A total 174 participants completed 100 trials at 500 ms duration. The subset of 121 participants in the processing speed analysis also completed the task at 70 ms, 90 ms, 125 ms and 200 ms. For participants who viewed all stimulus presentation times, a block consisted of 20 trials at each stimulus duration, with trial order randomized (100 trials in total for each duration, presented over five blocks).

### Visual integration task

The participants reported the left/right direction of coherently moving ‘signal’ dots within a display of randomly moving ‘noise’ dots. Display size, fixation, dot number, size and velocity were identical to the visual detection task, but contrast was fixed at 100%. Each dot had a lifespan of 1 s before reappearing in a new location (Figure 1b). Motion coherence was initially 100% and adjusted adaptively with two QUEST routines. Sensitivity (inverse threshold) was calculated as described above. A total 174 participants completed 100 trials at 750 ms duration. The subset of 121 participants in the processing speed analysis also completed the task at 100 ms, 200 ms, 2000 ms and 5000 ms.

### Auditory detection task

The participants reported whether a set of overlapping tones (50 on average), presented in pink noise at 50 dBA, was modulated upward or downward in frequency at 0.5 octaves per second. Each component had a random onset and a duration of 1 s (5 ms rise/fall ramps). Starting frequency was randomized between 500 and 4000 Hz. The compound stimulus was windowed to remove edge effects, resulting in a duration of 1 s (50 ms rise/fall ramps). Twenty-two trials at each of five different intensities (42, 44, 46, 51 and 57.5 dBA) were presented in a random order.

### Auditory integration task

The participants determined whether a set of concurrent, coherently modulated tones, in a background of incoherently modulated tones, was modulated upward or downward. Stimulus generation was as above, except tone life was 350 ms, and incoherently modulated tones were randomly assigned a direction of modulation <0.5 octaves per second. Stimuli were presented at 68 dBA. Twenty-two trials at each of five difficulty levels (15, 30, 45, 60 and 100% of tones coherently modulated) were presented in a random order.

### Simple response task

The continuous performance task consisted of 2.5 cm black arrows (2.5° visual angle), presented in a 5 × 5 cm light gray box (5° × 5° visual angle) on a medium gray background. Blocks consisted of 20 trials at each of nine different stimulus durations corresponding to the visual processing speed experiment mentioned below: 70, 90, 125, 200, 500, 750, 2000 and 5000 ms. Consistent with the visual motion tasks, the participants only reported direction and the trials were divided 50:50 left/right arrows with order of presentation randomized. A total of two blocks were presented each lasting 7 min, with participants allowed a break between blocks.

### General procedure

For visual tasks, the head position was stabilized at a distance of 57 cm with a chinrest in a dimly lit room (~140–160 Lux). Auditory stimuli were delivered via headphones. All the tasks were administered on a 15.4-inch computer screen (1440 Å~900 resolution at 60 Hz). The participants verbally reported their responses (left/right for motion tasks and simple response task, up/down for auditory detection and integration tasks) to a researcher, who entered the response via keyboard. The control participants were either tested in the same or similar rooms using identical equipment in a similar testing environment.

Before each task, the participants received instruction from experimenters and completed a brief practice block. During the tasks, the participants were provided with accurate feedback with a soft beep signaling incorrect responses. The participants were allowed breaks between test blocks.

All the collected data were analyzed with the software package SPSS Statistics 20 (IBM, Armonk, NY, USA). Group differences in IQ were assessed with a one-way analysis of variance and included as a covariate in all the subsequent analyses when significant (as indicated in each of the relevant sections of Supplementary Tables 1). In addition, to ensure that the observed statistical effects were not dependent on using IQ as a covariate, we also repeated all the analyses without covariates, and found the same overall pattern of results. The results differed between analyses in several minor cases, and these are noted in the text below. Handedness, gender and age were each found to not differ significantly for any of the analyses and were therefore not included as covariates.

## Results

### Visual detection and integration

#### Diagnostic group analyses

A multivariate analysis of covariance (ANCOVA) with dependent variables of motion integration performance (750 ms duration) and motion detection performance (500 ms duration) was used to assess differences between five different diagnostic groups (SSD, bipolar affective disorder, depression, other psychiatric diagnoses, healthy control—participant details in Supplementary Table 1) revealed a significant multivariate effect of diagnostic group on visual task performance, Pillai’s Trace=0.15, F(8,322)=3.20, *P*<0.01; *ŋ*_p_^2^=0.07.

Two follow-up univariate ANCOVAs were conducted separately on motion detection and integration data. The diagnostic groups differed significantly with integration F(4,161)=6.28, *P*<0.001; *ŋ*_p_^2^=0.14, but not detection F(4,161)=0.78, *P*=0.54. *Post hoc* paired Bonferroni corrected *t*-tests revealed that the effect of diagnosis on motion integration performance was driven by differences between the healthy control participants and patients with SSD (*P*<0.001, Cohen’s *d*=1.24) and bipolar affective disorder (*P*<0.01, Cohen’s *d*=1.23; Figure 1c). No other diagnostic groups differed significantly from one another.

#### Positive symptom analysis

We next tested the link between motion integration deficits and positive symptoms across diagnostic categories (participant details in Supplementary Table 1). Nonparametric Spearman correlation coefficients were calculated (PANSS positive symptom item ratings were not normally distributed). One-tailed *α*-values were used in accordance with the *a priori* prediction that more severe symptoms, as rated by the PANSS subscale, would be associated with poorer performance on the motion tasks (Table 2). Indeed, motion integration was negatively associated with the severity of delusions, conceptual disorganization, grandiosity, hostility and hallucinations, but not with excitement or suspiciousness. Motion detection was significantly associated only with grandiosity. All the significant correlations were negative, indicating that individuals with more severe positive symptoms performed worse on tasks of visual motion integration independent of diagnosis.

Furthermore, we also tested whether the observed negative correlations between positive symptom severity and visual motion integration deficits were also present in the inpatients who did not have an SSD diagnosis. This analysis revealed that even in this subset of participants, there was a significant negative relationship between the severity of visual motion integration deficits and both the delusions subscale (*ρ*=−0.37, *P*<0.01) and the conceptual disorganization subscale (*ρ*=−0.25, *P*<0.05). This suggests that the relationship between the severity of some positive symptoms and visual motion integration deficits was present even among those inpatients who did not have an SSD diagnosis. No other correlations were significant in this subset of participants.

#### Psychotic versus nonpsychotic analysis

**Figure 2:**
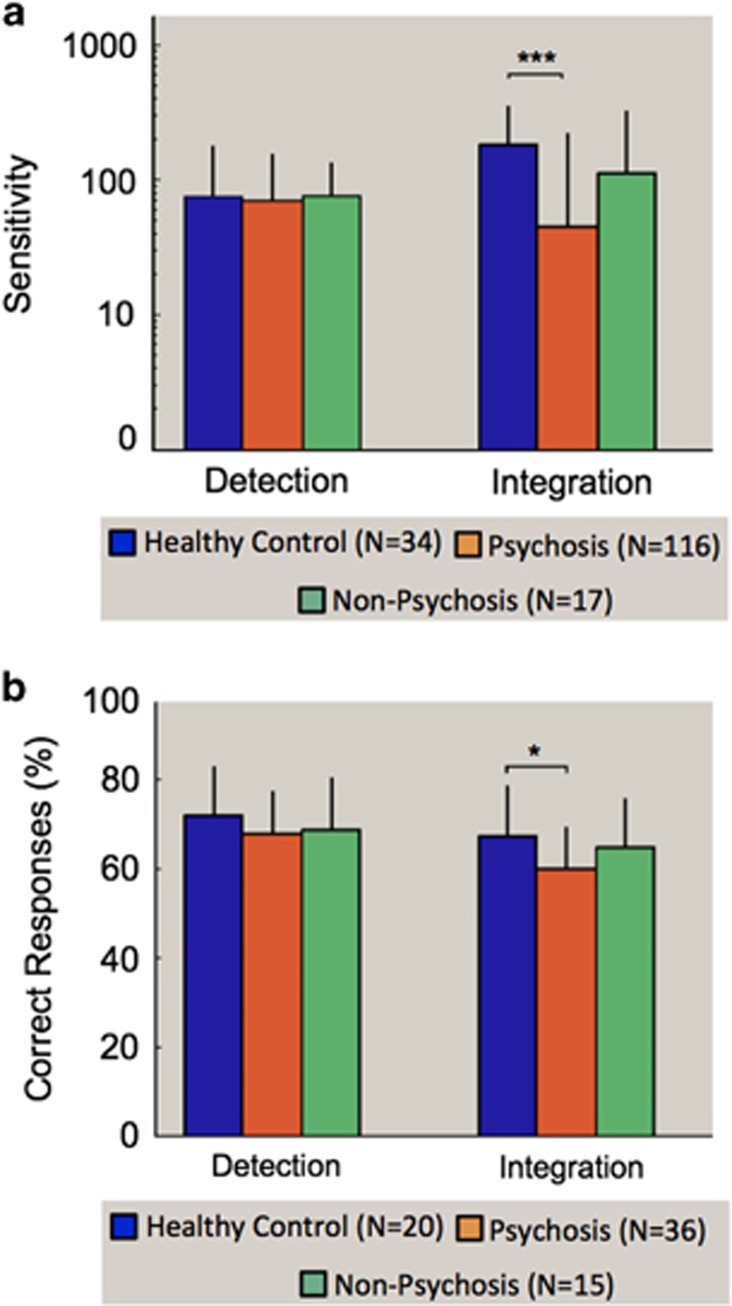
(**a**) Mean sensitivity for different psychosis groupings for visual detection and integration. (**b**) Mean percentage correct for different psychosis groups across auditory detection and integration tasks. Error bars represent 95% confidence intervals. Significance is denoted as follows: *P<0.05, ***P<0.001.

To further explore whether deficits in motion integration were related to psychosis, rather than diagnosis, we categorized patients as either psychotic or nonpsychotic according to criteria above (participant details in Supplementary Table 2). A multivariate ANCOVA with dependent variables of motion integration (750 ms duration) and motion detection (500 ms duration) performance revealed a significant multivariate effect of psychosis grouping on the visual tasks, Pillai’s Trace=0.12, F(4,326)=5.36, *P*<0.001; *ŋ*_p_^2^=0.06. Two follow-up univariate ANCOVAs were conducted separately on the detection and integration data. Diagnostic groups differed significantly in performance on motion integration, F(2,163)=10.93, *P*<0.001; *ŋ*_p_^2^=0.12, but not detection, F(2,163)=0.06, *P*=0.95. *Post hoc* paired Bonferroni corrected *t*-tests revealed that psychotic patients were impaired in coherent motion relative to healthy control participants (*P*<0.001), with a trend toward worse performance by psychotic than nonpsychotic patients (*P*=0.054; Figure 2a). When IQ was not included as a covariate, the group difference between psychotic and nonpsychotic patients was significant (*P*=0.04). Moreover, this contrast remained significant when patients with a diagnosis of an SSD were excluded from analysis (*P*<0.01), demonstrating that the difference in task performance between patients with psychosis and healthy control participants was not driven solely by patients with an SSD diagnosis.

To investigate whether a latent symptom structure underpinned the symptom associations with sensory integration, we used hierarchical cluster analysis with Ward’s method to classify the participants on the basis of their performance in the visual integration task (at the 750 ms stimulus duration because data were available for the greatest number of participants). A three-cluster solution was indicated, with cluster 3 of particular interest. This cluster contained exclusively participants classified as psychotic, across several different diagnoses. The participants in this cluster displayed markedly impaired visual integration relative to other clusters, but comparable performance in the motion detection task (see Supplementary Figures 1A and B). Moreover, membership of the high-impairment cluster was characterized by a greater severity of only conceptual disorganization and grandiosity but no other positive symptom (see Supplementary Figures 1C and D and Supplementary Table 3).

### Auditory detection and integration

#### Diagnostic group analyses

Two separate ANCOVAs were conducted to analyze the effect of diagnostic group (SSD, bipolar, depression, other psychiatric diagnosis, healthy control) on auditory detection and integration performance (participant details in Supplementary Table 1).

For auditory detection, ANCOVA revealed no significant effect of diagnostic group on task performance, F(4,60)=1.5, *P*=0.22. For auditory integration, there was a nonsignificant trend toward a main effect of diagnosis, F(4,60)=2.27, *P*=0.07, with comparable impairments seen in the SSD (Mean=59.75% correct, s.d.=7.06) and bipolar disorder groups (Mean=61.07%, s.d.=8.73) relative to healthy controls (Mean=67.42%, s.d.=10.98). As performance was quite poor on this task for all the groups, single-sample *t*-tests were conducted to rule out the possibility that a floor effect could account for the lack of significant difference between patient groups. Performance was found to be significantly above chance for all participants, as well as for all patient groups considered separately (*P*<0.05).

#### Psychotic versus nonpsychotic analysis

As similar deficits were seen across schizophrenia and bipolar disorder, the data were examined using the psychotic or nonpsychotic grouping used in the visual analysis (participant details in Supplementary Table 2). For auditory detection, ANCOVA showed no significant effect of psychosis grouping on task performance, F(2,62)=0.86, *P*=0.43. For auditory integration, performance significantly differed as a function of psychosis, F(2,62)=4.65, *P*<0.05; *ŋ*_p_^2^=0.13. *Post hoc* paired Bonferroni corrected *t*-tests revealed that psychotic patients were impaired in auditory integration relative to healthy control participants (*P*<0.05); however, if IQ was not covaried, the effect became a nonsignificant trend (*P*=0.052). All other comparisons were not significant (Figure 2b). Symptom correlation analysis was not run for auditory processing due to the relatively small sample size. Correlations between performance on the visual and auditory measures was similarly not obtained as the tasks were completed by independent samples.

### Control experiments and analysis

#### Visual processing speed

To determine whether impaired performance in visual integration reflected a deficit in processing speed, a subset of 121 participants was tested at multiple stimulus durations for both motion detection and motion integration (participant details in Supplementary Table 4). Two 5 × 5 mixed-design ANCOVAs were conducted separately for the motion detection and integration tasks to assess the effect of stimulus duration (coherent motion: 100, 200, 750, 2000, 5000 ms and detection: 70, 90, 125, 200, 500 ms) and diagnosis (SSD, bipolar affective disorder, major depression, other psychiatric diagnoses, healthy control). For both the analyses, Mauchly’s test indicated that the parametric assumption of sphericity was violated (*P*<0.001) and a Greenhouse–Geisser correction was therefore applied.

**Figure 3:**
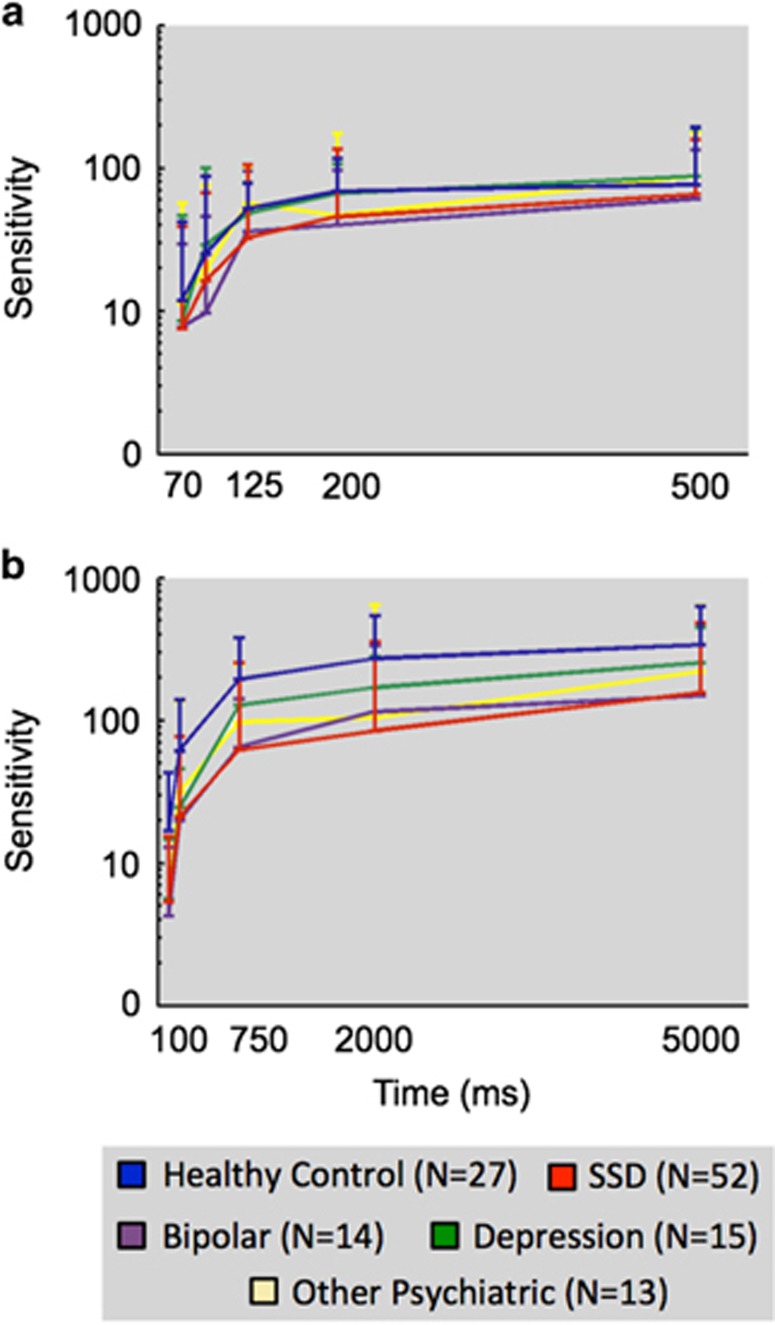
Mean sensitivity as a function of stimulus duration among different diagnostic groups for (**a**) visual detection and (**b**) visual integration tasks. Data are plotted on a logarithmic scale. Error bars represent 95% confidence intervals. SSD, schizophrenia spectrum disorder.

For motion detection, ANCOVA revealed a significant main effect of stimulus duration on motion sensitivity, F(3.06,339.76)=5.96, *P*<0.01; *ŋ*_p_^2^=0.05 but no main effect of diagnosis F(4,111)=1.81, *P*=0.13 (Figure 3a). In addition, a separate 2 × 5 ANCOVA revealed no significant differences in performance between participants who were assessed at multiple stimulus durations and those who were assessed only at 500 ms duration, F(1,157)=0.08, *P*=0.67, and no interaction between this factor and diagnosis, F(4,1577)=0.27, *P*=0.90.

In contrast an ANCOVA for coherent motion sensitivity revealed a significant main effect of diagnosis, F(4,115)=4.71, *P*<0.01; *ŋ*_p_^2^=0.14, and a significant main effect of stimulus duration, F(3.69,424.35)=5.57, *P*<0.001; *ŋ*_p_^2^=0.05, on coherent motion sensitivity. *Post hoc* paired Bonferroni corrected *t*-tests revealed that the main effect of diagnosis was driven by significant differences between healthy control participants and both patients with SSD (*P*<0.01) and patients with bipolar affective disorder (*P*<0.05). No other groups were significantly different from one another. There was no significant interaction between diagnosis and stimulus duration, F(14.76,424.32)=1.20, *P*=0.26, indicating that the different diagnostic groups did not vary in their pattern of impairment with increasing stimulus durations (Figure 3b).

#### Visual sustained attention

To assess whether the observed motion integration impairments were due to impaired sustained attention in the psychotic patients, a subset of 40 participants who completed visual motion tasks completed a simple response task designed to replicate the cognitive and attentional demands of our visual task (participant details in Supplementary Table 4). Error rates in the verbal report of arrow direction across all conditions and groups were very low (overall median=0.25%, range 0–4.5%), indicating that all patients were able to manage the cognitive and attentional demands of our visual tasks. We, therefore, calculated a single measure of total errors across all trials performed for each participant. There was no significant relationship between simple response task performance and either visual detection, *r*=−0.09, *P*=0.59, or visual integration, *r*=−0.17, *P*=0.30.

#### Medication

In some circumstances, antipsychotic or benzodiazepine medication were found to impair visual sensitivity.^30^ In the present study, antipsychotic dosage was greater in psychotic than in nonpsychotic inpatients, *t*(150)=−2.154, *P*<0.05, but the groups did not differ in benzodiazepine dosage, *t*(150)=1.01, *P*=0.31. We used linear regression to determine whether, among individuals receiving psychotropic medication, antipsychotic or benzodiazepine dosages predicted performance on visual tasks. There was no association between visual motion integration sensitivity and either antipsychotic dosage, *β*=−0.11, *P*=0.23 or benzodiazepine dosage, *β*=−0.18, *P*=0.14. Likewise, visual motion detection sensitivity was not associated with either antipsychotic dosage, *β*=−0.12, *P*=0.21, or benzodiazepine dosage, *β*=−0.17, *P*=0.18.

## Discussion

The present study assessed sensory detection and integration in a heterogeneous sample of psychiatric patients and healthy controls. Testing individuals across diagnostic groups showed that, contrary to previous claims,^13, 15, 18^ deficits in visual motion integration are not unique to schizophrenia. The deficits extended to other psychotic disorders, while nonpsychotic patients performed at levels similar to healthy controls. Furthermore, the degree of impairment in visual motion integration correlated with clinical ratings of positive symptoms, providing further evidence for an association between sensory integration impairment and psychosis. Importantly, the same pattern of intact sensory detection and impaired sensory integration using comparable dynamic stimuli was demonstrated for we believe the first time in the auditory domain.

Another major goal of this study was to examine factors such as reduced processing speeds or inattention as explanations for these and other results.^13, 18^ For example, it is known that in healthy populations, sensitivity for coherent motion increases with stimulus duration up to 2000–3000 ms, while contrast detection performance plateaus around 200–300 ms.^31^ The stimulus durations typically used (500–1000 ms) could saturate the detection but not the integration system, so a reduction in processing speed could have explained the previous findings of deficits being limited in the integration tasks. However, our results show that a similar degree of deficit for patients exists across different stimulus durations with all groups plateauing in performance at similar time points (Figure 3), excluding this possibility.

Attentional impairments occur across a range of psychiatric disorders^32, 33, 34^ and are well established in populations with schizophrenia.^35, 36^ We were, therefore, mindful that patients might have an increased potential to disengage from our sensory tasks confounding the interpretation of any deficits observed. To mitigate the impact of this potential confound, we asked all the participants to respond verbally rather than through button press. This allowed the experimenters to detect clear instances when the participants had disengaged from the experiment and would recommend a longer break at the completion of the testing block (all participants were provided breaks between each block of testing). To ensure that participants were indeed able to maintain task focus within the individual testing blocks using this verbal report design, we ran an additional assessment in a subset of our participants on a continuous performance task involving equivalent trial durations and response reporting requirements. Patients performed well on this task with no significant relationship between results for the attentional task and those for either visual detection or integration. These findings are consistent with other previous studies^15^ and show that our results cannot be accounted for by an inability for patients to maintain focus on the tasks. Furthermore, as the contrast detection and coherent motion tasks involved identical instructions (‘report whether the dots are moving to the left or right’), we can similarly rule out any general difference in cognitive load requirements between the two tasks. It is important to note, however, that while all participants had normal or corrected-to-normal vision/audition, and our detection tasks provided an indirect measure of acuity, we cannot exclude the possibility of undetected differences in sensory acuity. Any disparities could have minimized between-group differences among patient groups and/or magnified patient control group differences.^37^ Finally, our medication analyses showed no significant association of antipsychotic or benzodiazepine drugs on the visual task performance.

Having considered these potential confounds, our finding of impaired sensory integration—but not sensory detection—in both vision and audition provides some important clues to potential biological mechanisms associated with psychosis. The hierarchical nature of visual and auditory processing permits the design of stimuli that discretely target different components of the pathway with successive involvement of integrative processes. In vision, the neural coding of contrast probably occurs at the earliest stages of the visual pathway.^38, 39^ The responses of individual V1 neurons vary with stimulus contrast over relatively small areas of the visual field, indicating this visual feature is coded during the largely parallel processing that occurs at, or before V1. In contrast, the processing of coherent motion involves visual cortical area V5/MT,^40, 41^ where the individual motion signals from V1 are received and integrated.^42^ In audition, there are some similarities, where neurons in the monkey primary auditory cortex (A1) respond selectively to pure tones within a specific frequency range^43^ and neurons in the lateral auditory belt respond more strongly to frequency-modulated sweeps.^44^ As a consequence, our findings indicate that the external visual and auditory information is being received and transmitted to the primary sensory cortices with an apparent intensity and quality sufficient for normal contrast sensitivity function. It is only at the point when the individual neural signals are integrated across populations of larger neural assemblies that deficits become apparent. Such deficits in neural integration are likely to be relevant to increasingly higher-level cognitive and perceptual tasks that involve large complexes of neurons and synapses.

These results are consistent with many of the leading biological models of schizophrenia implicating neural abnormalities with diffuse brain-wide consequences, such as altered brain structure,^45^ neural oscillation patterns^20^ or neurotransmitter function.^22^ However, our findings that the functional deficits exist beyond schizophrenia suggest the relevant biological abnormalities may similarly underlie positive symptoms beyond traditional diagnostic boundaries. This conclusion is in line with recent cognitive, genetic, neuroimaging and biomarker findings^46, 47, 48^ but importantly, using a different battery of tasks, extends these transdiagnostic findings into the domain of perception and therefore represents a new and independent finding. Concordantly, the identification of a subset of psychotic patients characterized by high levels of conceptual disorganization and severely impaired visual integration using a dynamic display, is consistent with an earlier finding of an association between conceptual disorganization and impaired perceptual organization using static visual stimuli.^49^

This transdiagnostic finding also accords with the concept that psychosis is syndromal and its sensory integration pathology is not contingent on psychiatric diagnosis. Accordingly, the current findings are consistent with the effects of the psychotomimetic hallucinogen psilocybin, which was similarly found to selectively impair visual integration but not sensory detection in population of healthy participants.^50^

Although we were successful in assessing a range of different measures across a large participant group, we were constrained by time demands on participants. As a consequence, because we posited that disrupted sensory integration may contribute to positive symptoms, we restricted our patient assessments to focus on this symptom cluster. It therefore remains possible that a similar relationship exists between integrative deficits and other negative and cognitive symptoms that are also exhibited across a range of psychotic disorders.^23^ One further point worth future investigation is the extent to which the findings here are limited to the acute state of illness, due to our recruitment from an acute psychiatry inpatient unit. As previous studies suggest that sensory integration impairments may be related to core dimensions of functioning that are potentially distinct from psychosis but covary with it,^51^ future research should interrogate specific temporal relationships between symptomatology and integration deficits in more detail.

Finally, it is important to note that a history of substance abuse was an exclusion criteria for our controls, but not the patient samples given the very high rates of substance abuse in acute adult inpatient cohorts. Therefore, although patients were only tested after any intoxication or withdrawal had resolved and those with a primary substance use diagnosis were excluded, substance-related effects remain a possible confound. In the future, the impact of external factors including individual differences in tobacco and caffeine consumption should be considered in studies investigating the relationship between symptom profiles and sensory integration performance.

Together, these findings are consistent with a dimensional, rather than categorical, view of psychotic illnesses. Moreover, these data suggest that psychosis as a syndrome may share a similar disruption of sensory integration across disorders and as such does not provide diagnostic specificity. Future assessment of sensory abnormalities in this field ought to include participants with a range of psychiatric diagnoses to more finely delineate whether differences in sensory impairment exist between different disorders or whether they reflect symptom specific features that span a range of psychotic illnesses.

## Figures and Tables

**Table 1:** Overall demographic and clinical characteristics

*Characteristic*	*Schizophrenia spectrum disorders*	*Bipolar affective disorder*	*Major depression*	*Other psychiatric diagnoses*	*Healthy control*
*N* (Total)	98^a^	35^b^	31^c^	31^d^	54
Male	67	19	19	17	31
					
*Handedness*					
Right	80	32	24	23	51
Left	7	0	3	3	4
Mixed	11	3	3	5	
					
	*Mean (s.d.)*	*Mean (s.d.)*	*Mean (s.d.)*	*Mean (s.d.)*	*Mean (s.d.)*
Age (years)	37.6 (10.8)	42.8 (13.1)	35.6 (11.3)	34.0 (13.2)	41.5 (15.7)
NART IQ estimate^e^	102.6 (9.6)	105.7 (12.0)	105.1 (9.8)	100.2 (11.5)	111.3 (8.3)
Antipsychotic daily (mg)	426.8 (327.2)	330.6 (240.8)	132.8 (201.9)	169.3 (212.7)	NA
Benzodiazepine daily (mg)	10.1 (20.8)	14.1 (19.9)	16.3 (26.1)	6.8 (8.4)	NA
					
*Participant numbers by analysis*	*Schizophrenia spectrum disorders*	*Bipolar affective disorder*	*Major depression*	*Other psychiatric diagnoses*	*Healthy control*
Vision: diagnostic group analysis	71	23	19	20	34
Vision: symptom correlation analysis	47	22	14	13	0
Audition: diagnostic group analysis	22	8	10	11	20
Vision: processing speed analysis	52	14	15	13	27
Vision: simple response task	20	10	6	4	0
Medication analysis	72	24	15	19	0
			
*Participant numbers by analysis*	*Psychosis*	*Nonpsychotic inpatients*		*Healthy control*	
Vision: psychosis analysis	116	17		34	
Audition: psychosis analysis	36	15		20	

Abbreviations: IQ, intelligence quotient; NA, not available; NART, National Adult Reading Test.

a Including 70 with schizophrenia, 27 with schizoaffective disorder and 1 with schizophreniform psychosis.

b Including 28 in a manic episode at the time of testing and 7 in a depressed episode.

c Including 2 patients with a diagnosis of major depression with psychotic features.

d Including 9 patients with borderline personality disorder; 8 with first-episode psychosis; 4 with brief psychotic episode; 2 with delusional disorder; 2 with drug-induced psychosis; 1 with each of factitious disorder, delirium and posttraumatic stress disorder; and 3 participants admitted to the inpatient unit following a situational crisis.

e Due to dyslexia or illiteracy, IQ estimates were not available for 6 individuals in the schizophrenia group, 2 individuals in the bipolar affective disorder group and 1 participant in the other psychiatric diagnosis group. In addition, 2 healthy control participants did not complete the NART.

**Table 2:** Correlations between visual impairments and positive symptom

*PANSS subscale*	*Motion detection (500* *ms)*	*Motion integration (750* *ms)*
Delusions	−0.00	−0.27^a^
Conceptual disorganization	0.14	−0.24^b^
Hallucinations	0.07	−0.17^b^
Excitement	0.06	0.01
Grandiosity	−0.19^b^	−0.28^a^
Suspiciousness	−0.16	−0.10
Hostility	−0.16	−0.25^a^

a *α*<0.01.

b *α*<0.05.

Spearman correlation coefficients and significance levels (one-tailed) show the relationship between the Positive and Negative Syndrome Scale (PANSS) positive symptom subscale items and visual local and global motion sensitivity.
