# Supplementary material for: Sensory integration deficits support a dimensional view of psychosis and are not limited to schizophrenia
Source: Transl Psychiatry. 2017 May 9;7(5):e1118–. doi: 10.1038/tp.2017.69 (PMC5534945; doi:10.1038/tp.2017.69)
Supplement: Supplementary Information [file tp201769x1.docx]

TABLE 1: Participant characteristics for the diagnostic and symptom analyses

| **Vision - Diagnostic Analysis** | Schizophrenia Spectrum Disorders | Bipolar Affective Disorder | Major Depression | Other Psychiatric Diagnoses | Healthy Control |
| --- | --- | --- | --- | --- | --- |
| N (Total) | 71^a^ | 23^b^ | 19^c^ | 20^d^ | 34 |
| Male | 49 | 13 | 10 | 9 | 20 |
| Handedness  Right  Left  Mixed | 60  4  7 | 21  0  2 | 16  2  1 | 17  2  1 | 32  2  0 |
|  | Mean (SD) | Mean (SD) | Mean (SD) | Mean (SD) | Mean (SD) |
| Age (years) | 37.3 (10.5) | 43.1 (14.2) | 36.4 (11.0) | 30.6 (10.7) | 39.6 (16.0) |
| NART IQ Estimate† | 102.8 (10.3) | 105.4 (13.6) | 106.8 (10.3) | 97.7 (11.5) | 111.9 (8.6) |
| Antipsychotic Daily [mg] | 430.3 (333.6) | 340.1 (218.8) | 129.8 (206.9) | 155.0 (207.7) | N/A |
| Benzodiazepine Daily [mg] | 10.5 (21.4) | 13.5 (18.8) | 16.6 (26.8) | 6.7 (8.6) | N/A |
| **Vision - Symptom Analysis** | Schizophrenia Spectrum Disorders | Bipolar Affective Disorder | Major Depression | Other Psychiatric Diagnoses | Healthy Control |
| *n* | 47^e^ | 22^f^ | 14^g^ | 13^h^ | N/A |
| Male | 33 | 12 | 7 | 6 | N/A |
| Handedness  Right  Left  Mixed | 39  3  5 | 21  0  1 | 13  1  0 | 11  1  1 |  |
|  | Mean (SD) | Mean (SD) | Mean (SD) | Mean (SD) | Mean (SD) |
| Age (years) | 38.8 (10.4) | 42.1 (13.0) | 39.4 (10.8) | 28.0 (10.1) | N/A |
| NART IQ Estimate‡ | 103.3 (9.1) | 106.3 (12.6) | 106.5 (10.1) | 97.3 (11.2) | N/A |
| Antipsychotic Daily [mg] | 542.8 (314.1) | 389.1 (222.7) | 189.7 (218.9) | 216.4 (217.2) | N/A |
| Benzodiazepine Daily [mg] | 10.3 (24.8) | 15.7 (21.6) | 16.8 (30.6) | 5.6 (7.2) | N/A |
| **Audition - Diagnostic Analysis** | Schizophrenia Spectrum Disorders | Bipolar Affective Disorder | Major Depression | Other Psychiatric Diagnoses | Healthy Control |
| N (Total) | 22^i^ | 8^j^ | 10^k^ | 11^l^ | 20 |
| Male | 14 | 5 | 8 | 8 | 10 |
| Handedness  Right  Left  Mixed | 16  2  4 | 7  1  0 | 7  1  2 | 6  1  4 | 18  2  0 |
|  | Mean (SD) | Mean (SD) | Mean (SD) | Mean (SD) | Mean (SD) |
| Age (years) | 35.9 (10.0) | 41.8 (13.2) | 33.7 (12.7) | 42.1 (14.8) | 44.6 (15.1) |
| NART IQ Estimate * | 101.1 (6.3) | 105.5 (7.7) | 102.5 (9.1) | 105.3 (10.2) | 110.3 (7.8) |
| Antipsychotic Daily [mg] | N/A | N/A | N/A | N/A | N/A |
| Benzodiazepine Daily [mg] | N/A | N/A | N/A | N/A | N/A |

† IQ differed between groups *F*(4, 162) = 6.77, *p* < .001, and was included as a covariate in analysis

‡ IQ did not differ significantly between groups, *F*(3, 86) = 2.31, *p* = .08. Due to dyslexia or illiteracy, IQ estimates were not available for 3 individuals in the schizophrenia group, 2 individuals in the bipolar affective disorder group, and 1 participant in the other psychiatric diagnosis group.

* IQ differed between groups *F*(4, 61) = 3.57, *p* < .05, and was included as a covariate in analysis. Due to dyslexia or illiteracy, IQ estimates were not available for 3 individuals in the schizophrenia group. In addition, 2 healthy control participants did not complete the NART.

^a^ Including 50 with schizophrenia, 20 with schizoaffective disorder, 1 with schizophreniform psychosis. ^b^ Including 20 in manic and 3 in depressed phase at testing. ^c^ Including 1 patient with major depression with psychotic features. ^d^ Including 7 patients with borderline personality disorder, 6 with first episode psychosis, 2 each with delusional disorder and drug-induced psychosis, 1 with factitious disorder, 2 admitted following situational crisis. ^e^ Including 34 with schizophrenia, 12 with schizoaffective disorder, 1 with schizophreniform psychosis. ^f^ Including 18 in manic and 4 in depressed phase at testing. ^g^ Including 1 patient with a diagnosis of major depression with psychotic features. ^h^ Including 6 patients with first episode psychosis, 5 with borderline personality disorder, 1 with drug-induced psychosis, 1 admitted following situational crisis. ^i^ Including 16 with schizophrenia, 6 with schizoaffective disorder. ^j^ Including 6 in manic and 2 in depressed phase at testing. ^k^ Including 1 patient with diagnosis of major depression with psychotic features. ^l^ Including 4 patients with brief psychotic episode, 2 with borderline personality disorder, 1 with each of delirium, first episode psychosis, post-traumatic stress disorder, 1 admitted following situational crisis.

TABLE 2: Participant characteristics for psychosis analyses

| **Vision - Psychosis Analysis** | Psychosis | Non-psychotic Inpatients | Healthy Control |
| --- | --- | --- | --- |
| *n* | 116 | 17 | 34 |
| Male | 70 | 11 | 20 |
| Breakdown of diagnosis |  |  |  |
| Schizophrenia Spectrum Disorders | 71 | 0 | N/A |
| Bipolar Affective Disorder | 19 | 4 | N/A |
| Major Depression | 9 | 10 | N/A |
| Other Psychiatric Diagnoses | 17 | 3 | N/A |
| Handedness  Right  Left  Mixed | 99  6  11 | 15  2  0 | 32  2  0 |
|  | Mean (SD) | Mean (SD) | Mean (SD) |
| Age (years) | 37.0 (11.5) | 38.5 (13.6) | 39.6 (16.0) |
| NART IQ Estimate ^+^ | 102.7 (11.0) | 105.7 (13.4) | 111.9 (8.6) |
| PANSS Positive Subscale | 19.89 (4.62) | 9.92 (2.32) | N/A |
| Antipsychotic Daily [mg] | 349.8 (311.7) | 199.1 (252.1) | N/A |
| Benzodiazepine Daily [mg] | 10.5 (19.1) | 17.2 (28.1) | N/A |
| **Audition - Psychosis Analysis** | Psychosis | Non-psychotic Inpatients | Healthy Control |
| *n* | 36 | 15 | 20 |
| Male | 22 | 13 | 10 |
| Breakdown of diagnosis |  |  |  |
| Schizophrenia Spectrum Disorders | 22 | 0 | N/A |
| Bipolar Affective Disorder | 3 | 5 | N/A |
| Major Depression | 3 | 8 | N/A |
| Other Psychiatric Diagnoses | 8 | 2 | N/A |
| Handedness  Right  Left  Mixed | 25  3  8 | 11  1  3 | 18  2  0 |
|  | Mean (SD) | Mean (SD) | Mean (SD) |
| Age (years) | 37.6 (10.9) | 37.5 (15.3) | 44.6 (15.1) |
| NART IQ Estimate ‡ | 101.7 (7.4) | 106.0 (9.1) | 110.3 (7.8) |
| PANSS Positive Subscale | 16.08 (3.76) | 9.00 (2.20) | N/A |
| Antipsychotic Daily [mg] | N/A | N/A | N/A |
| Benzodiazepine Daily [mg] | N/A | N/A | N/A |

^+^ IQ differed between groups, *F*(2,164) = 9.52, *p* < .001, and was included as a covariate in analysis.

‡ IQ differed between groups *F*(2,63) = 7.09, *p* < 0.01, and was included as a covariate in analysis.

Table 3. Cluster characteristics with respect to diagnosis, psychosis, and symptom ratings.

| **Cluster** | **1** | **2** | **3** | ***χ^2^*** | ***p*** |
| --- | --- | --- | --- | --- | --- |
| Schizophrenia Spectrum Disorders | 48 | 17 | 10 | 30.15 | < .001 |
| Bipolar Affective Disorder | 17 | 7 | 3 | - | - |
| Major Depression | 11 | 8 | 1 | - | - |
| Other Psychiatric Diagnoses | 10 | 10 | 1 | - | - |
| Healthy Control | 9 | 25 | 0 | - | - |
| **Cluster** | **1** | **2** | **3** | ***χ^2^*** | ***p*** |
| Psychotic | 76 | 33 | 15 | 28.47 | < .001 |
| Non-psychotic | 10 | 9 | 0 | - | - |
| Healthy control | 9 | 25 | 0 | - | - |
| **Cluster** | **1** | **2** | **3** | ***F*** | ***p*** |
| Delusions | 3.66 | 2.67 | 3.83 | 3.99 | < .05 |
| Conceptual Disorganisation | 1.81 | 1.46 | 2.50 | 4.65 | < .05 |
| Hallucinations | 2.68 | 2.21 | 2.67 | 0.69 | .50 |
| Excitement | 2.31 | 2.38 | 2.25 | 0.05 | .95 |
| Grandiosity | 2.86 | 2.08 | 3.42 | 3.61 | < .05 |
| Suspiciousness | 3.39 | 2.92 | 2.83 | 1.42 | .25 |
| Hostility | 2.53 | 1.88 | 2.33 | 1.30 | .28 |
| PANSS Positive Total | 19.24 | 15.58 | 19.83 | 4.49 | < .05 |

***χ^2^*** statistics refer to results of a chi-square test of independence assessing the null hypothesis that cluster membership simply reflects the relative proportions of diagnoses and psychosis states. *F* statistics refer to the results of three-way univariate ANOVAs with cluster membership as a between-participants factor.

TABLE 4: Vision – Processing speed and sustained attention analysis

| **Processing Speed** | Schizophrenia Spectrum Disorders | Bipolar Affective Disorder | Major Depression | Other Psychiatric Diagnoses | Healthy Control |
| --- | --- | --- | --- | --- | --- |
| N (Total) | 52^a^ | 14^b^ | 15 | 13^c^ | 27 |
| Male | 35 | 8 | 9 | 6 | 16 |
| Handedness  Right  Left  Mixed | 42  4  6 | 12  0  2 | 12  2  1 | 11  1  1 | 25  2  0 |
|  | Mean SD | Mean SD | Mean SD | Mean SD | Mean SD |
| Age (years) | 36.9 10.9 | 43.9 13.7 | 34.2 10.1 | 30.6 10.6 | 37.2 16.0 |
| NART IQ Estimate^e^ † | 103.5 10.2 | 106.6 13.8 | 107.5 9.9 | 95.9 10.7 | 111.2 9.0 |
| Antipsychotic Daily [mg] | 381.0 332.7 | 259.8 246.8 | 106.0 180.8 | 164.2 248.7 | N/A |
| Benzodiazepine Daily [mg] | 8.7 18.8 | 11.8 19.6 | 11.0 13.1 | 7.9 9.2 | N/A |
| **Sustained Attention** | Schizophrenia Spectrum Disorders | Bipolar Affective Disorder | Major Depression | Other Psychiatric Diagnoses | Healthy Control |
| *n* | 20^d^ | 10^e^ | 6 | 4 ^f^ | 0 |
| Male | 15 | 7 | 3 | 2 | N/A |
| Handedness  Right  Left  Mixed | 15  2  3 | 9  0  1 | 5  1  0 | 4  0  0 | N/A  N/A  N/A |
|  | Mean SD | Mean SD | Mean SD | Mean SD | Mean SD |
| Age (years) | 37.0 11.0 | 41.1 12.9 | 40.5 10.5 | 25.0 9.3 | N/A N/A |
| NART IQ Estimate‡ | 104.1 8.2 | 108.3 12.6 | 106.7 7.7 | 92.7 2.8 | N/A N/A |
| Antipsychotic Daily [mg] | 598.1 335.3 | 408.2 266.9 | 201.7 248.9 | 457.6 207.8 | N/A |
| Benzodiazepine Daily [mg] | 8.3 26.7 | 19.0 26.9 | 10.8 15.9 | 9.5 8.2 | N/A |

† IQ differed between groups, *F*(4, 116) = 5.40, *p* < .01, and was included as a covariate in analysis.

‡ IQ did not differ between groups, *F*(3,32) = 2.24,  *p* =.10. Due to dyslexia or illiteracy, IQ estimates were not available for 2 individuals in the schizophrenia group, 1 individual in the bipolar affective disorder group, and 1 participant in the psychiatric-other group.

^a^ Including 37 with schizophrenia and 15 with schizoaffective disorder.

^b^ Including 13 in a manic episode at time of testing and 1 in a depressed episode.

^c^ Including 3 patients with first episode psychosis, 5 with borderline personality disorder, 2 with delusional disorder, 1 with each of drug-induced psychosis and factitious disorder and 1 participant admitted to the inpatient unit following a situational crisis.

^d^ Including 16 with schizophrenia, and 4 with schizoaffective disorder.

^e^ Including 10 in a manic episode at time of testing.

^f^ Including 3 patients with first episode psychosis and 1 with borderline personality disorder.


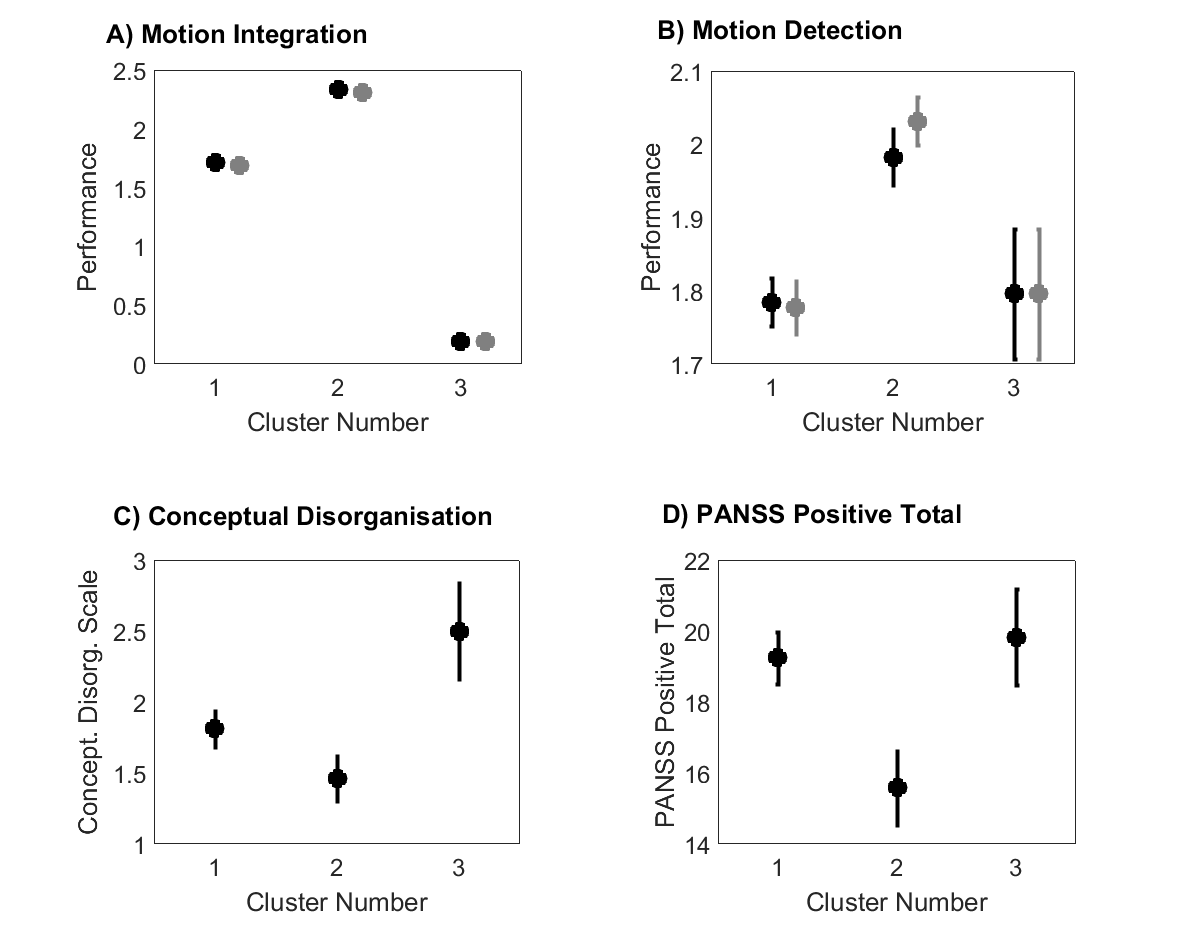


Figure 1. Mean task performance and symptom characteristics as a function of cluster membership. Black markers: all participants. Grey markers: healthy control participants excluded. Error bars represent SEM. (A) Performance on the motion integration task at 750ms stimulus duration. (B) Performance on the motion detection task at 500ms stimulus duration. (C) Conceptual disorganisation ratings. (D) Total ratings for PANSS positive subscale.
